# Novel flaviviruses from mosquitoes: Mosquito-specific evolutionary lineages within the phylogenetic group of mosquito-borne flaviviruses

**DOI:** 10.1016/j.virol.2014.07.015

**Published:** 2014-09

**Authors:** Eili Huhtamo, Shelley Cook, Gregory Moureau, Nathalie Y. Uzcátegui, Tarja Sironen, Suvi Kuivanen, Niina Putkuri, Satu Kurkela, Ralph E. Harbach, Andrew E. Firth, Olli Vapalahti, Ernest A. Gould, Xavier de Lamballerie

**Affiliations:** a Department of Virology, Haartman Institute, Faculty of Medicine, University of Helsinki, Helsinki, Finland; b Department of Life Sciences, Natural History Museum, Cromwell Road, London SW7 5BD, United Kingdom; c UMR D 190 “Emergence des Pathologies Virales”, Aix Marseille University, IRD French Institute of Research for Development, EHESP French School of Public Health, 27 Boulevard Jean Moulin, Marseille 13005, France; d Department of Virology and Immunology, Helsinki University Central Hospital Laboratory (HUSLAB), P.O. Box 400, Haartmaninkatu 3, 00029 HUS, Helsinki, Finland; e Division of Virology, Department of Pathology, University of Cambridge, Cambridge CB2 1QP, United Kingdom; f Division of Microbiology and Epidemiology, Department of Basic Veterinary Sciences, University of Helsinki, Helsinki, Finland

**Keywords:** Flavivirus, Isolate, Mosquito, Complete genome, Phylogenetic analysis

## Abstract

Novel flaviviruses that are genetically related to pathogenic mosquito-borne flaviviruses (MBFV) have been isolated from mosquitoes in various geographical locations, including Finland. We isolated and characterized another novel virus of this group from Finnish mosquitoes collected in 2007, designated as Ilomantsi virus (ILOV). Unlike the MBFV that infect both vertebrates and mosquitoes, the MBFV-related viruses appear to be specific to mosquitoes similar to the insect-specific flaviviruses (ISFs). In this overview of MBFV-related viruses we conclude that they differ from the ISFs genetically and antigenically. Phylogenetic analyses separated the MBFV-related viruses isolated in Africa, the Middle East and South America from those isolated in Europe and Asia. Serological cross-reactions of MBFV-related viruses with other flaviviruses and their potential for vector-borne transmission require further characterization. The divergent MBFV-related viruses are probably significantly under sampled to date and provide new information on the variety, properties and evolution of vector-borne flaviviruses.

## Highlights

• Mosquito-borne flavivirus-related viruses were isolated from Finnish mosquitoes.
• Isolates were reactive with flavivirus antibodies but appeared mosquito-specific.
• Sequence analysis identified related viruses from different parts of the world.
• These viruses represent unique properties among the mosquito-borne flavivirus group.

## Introduction

Members of the genus *Flavivirus*, family *Flaviviridae* are enveloped viruses that have a positive-sense single-stranded RNA genome. The flaviviral genome contains a single open-reading frame encoding a large polyprotein that is cleaved and processed by viral and host enzymes to form the mature structural proteins found in virions, the capsid (C), membrane (M) and envelope (E). In infected cells, seven non-structural viral proteins have been identified (NS1, NS2A, NS2B, NS3, NS4A, NS4B and NS5) (Chambers et al., 1990a, Pletnev et al., 2011). Although flaviviruses show considerable conservation of their genome organization, they exhibit divergent host ranges. In general, the flavivirus groups are phylogenetically relatively closely related and have associations with specific vector and/or vertebrate hosts (Cook and Holmes, 2006; Gaunt et al., 2001, Grard et al., 2007, Grard et al., 2010). The mosquito-borne flaviviruses (MBFVs) are the largest group with currently over 20 recognized species that include some of the most important pathogens of human arboviral diseases. The MBFVs can be divided into two main groups based on their mosquito-vector associations (Gaunt et al., 2001). The flaviviruses transmitted by *Stegomyia* mosquito species, which include yellow fever virus (YFV) and dengue virus (DENV), have life cycles involving various vertebrate hosts, including primates. The flaviviruses transmitted by *Culex* mosquito species include West Nile virus (WNV), Japanese encephalitis virus (JEV) and St Louis encephalitis virus (SLEV), which are characteristically maintained in life cycles involving birds. Humans may be incidentally infected but are generally considered to be dead-end hosts. Some viruses that are genetically relatively closely related to YFV appear to have no known arthropod vectors, *e.g.* Entebbe bat virus (ENTV) and Yokose virus (YOKV), and it has been proposed that they may have lost this vector-dependence (Kuno et al., 1998).

The flaviviruses transmitted by ticks are associated either with small mammals or seabirds and include pathogens that infect humans, such as tick-borne encephalitis virus (TBEV). In addition to flaviviruses that are hosted by both vertebrates and arthropods, other flaviviruses are defined as no-known vector (NKV) viruses. These viruses are at present considered to be hosted exclusively by small mammals and include viruses associated with bats, such as Entebbe bat virus (ENTV) and Rio Bravo virus (RBV), and viruses associated with rodents, such as Modoc virus (MODV). Additionally, another group of flaviviruses that has been characterized in more recent years, the insect-specific flaviviruses (ISFs) are currently known to infect only insect hosts, primarily mosquitoes. These viruses include cell fusing agent virus (CFAV) (Cammisa-Parks et al., 1992, Stollar and Thomas, 1975), Kamiti River virus (KRV) (Crabtree et al., 2003, Sang et al., 2003) and many recently identified related viruses from different regions of the world (Cook et al., 2006, Cook et al., 2009, Cook et al., 2012, Crabtree et al., 2009, Farfan-Ale et al., 2009, Hoshino et al., 2007, Hoshino et al., 2009, Huhtamo et al., 2012, Kim et al., 2009, Morales-Betoulle et al., 2008). Interestingly, some of these ISFs appear to be capable of integrating their genomic sequences into mosquito genomes (Crochu et al., 2004). The additional flaviviruses, Tamana bat virus (TABV) (de Lamballerie et al., 2002) and Ngoye virus (Grard et al., 2006) appear to represent highly divergent genetic lineages not closely associated with any currently recognized flavivirus group.

Until recently, all flavivirus genomes were considered to contain a single ORF encoding the viral proteins. However, it has now been shown that through a ribosomal frameshifting mechanism, an alternative-sized NS1 protein (NS1′) is produced by some mosquito-borne flaviviruses within the Japanese encephalitis virus group (Blitvich et al., 1999, Firth and Atkins, 2009). Also, an additional protein designated “fifo”, encoded as an overlapping ORF in the NS2A/NS2B coding sequence, has been detected in some insect-specific flaviviruses (Firth et al., 2010). Whereas the NS1′ protein has been associated with pathogenic properties (Melian et al., 2010), the possible functions of “fifo” are currently unknown.

Recently, six novel flaviviruses isolated from mosquitoes were published and shown to be genetically related to the taxonomically recognized mosquito-borne flaviviruses (MBFVs) (Pletnev et al., 2011), namely Nounané virus (NOUV) (Junglen et al., 2009) from Côte d’Ivoire, Chaoyang virus (CHAOV) from China and South Korea (Lee et al., 2013, Wang et al., 2009), Lammi virus (LAMV) from Finland (Huhtamo et al., 2009), Marisma mosquito virus (MMV) from Spain (Vazquez et al., 2012), Nanay virus (NANV) from Peru (Evangelista et al., 2013) and Barkedji virus from Senegal and Israel (Kolodziejek et al., 2013).

Crucially, these viruses do not appear to infect vertebrate cells readily despite their apparent similarity to MBFVs, the latter which readily infect vertebrate hosts. Here we review the available information for the currently-known MBFV-related viruses and report the isolation and characterization of four strains of LAMV and four strains of a potentially novel virus species tentatively named Ilomantsi virus (ILOV).

## Results

### Virus isolation, identification and sequence analysis

Homogenates of the mosquito pools were tested in 68 virus isolation attempts, in parallel, on C6/36 and Vero cells. From these, eight virus isolation cultures from C6/36 cells were identified as positive for flavivirus antigen in IFA whereas the Vero cells infected with the same mosquito homogenates remained negative. These viral isolates caused mild CPE (rounding of cells and increasing numbers of floating cells) on infected C6/36 cells, approximately one week post-infection. A fragment of the NS3 gene was amplified and sequenced from each isolate. The derived sequences (536 bp) identified the isolates as flaviviruses representing two distinct groups, the isolates M0727, M0719, S0739 and M0728 were identical to each other and shared 98.6% nucleotide homology with the prototype strain of LAMV (FJ606789, corresponding to nucleotides 5104-5639). Following the criterion of defining a flavivirus species based on nucleotide sequence comparisons (Kuno et al., 1998), these isolates were considered strains of LAMV as they shared over 84% pairwise nucleotide homologies with LAMV prototype virus. The second group of isolates was also identical to each other, including isolates M0724, M0720, M077 and M0726. These isolates shared only 67.3% nucleotide homology with the prototype strain of LAMV, and 67.1% with the LAMV 2007 strains and were considered to represent a separate flavivirus species provisionally designated Ilomantsi virus (ILOV) based on the mosquito collection site.

One representative strain from each group, namely (M0719) designated Lammi virus strain Mekrijärvi 2007 (LAMV-M07) and ILOV (M0724), was chosen for further analysis. Electron microscopy was performed on concentrated ILOV samples, which showed spherical flavivirus-like virions of approximately 40–50 nm in diameter (not shown). The complete coding sequences of LAMV-M07 and ILOV demonstrated the characteristic organization typical of flavivirus genomes, encoding a long polyprotein. The coding sequence of ILOV was 10,353 bp, encoding a 3451 amino acid polyprotein (Genbank accession KC692067). Within the complete coding sequence, ILOV and LAMV were found to share only 62.7% pairwise identity at the nucleotide level and 64% at the amino acid level, demonstrating that they were separate virus species (Kuno et al., 1998, Pletnev et al., 2011). The ORF sequence of LAMV-M07 was 10,302 bp, encoding a polyprotein of 3434 amino acids (GenBank accession KC692068). The LAMV-M07 strain shared 98.8% homology at the nucleotide level and 99.6% at the amino acid level with the LAMV prototype strain. Similar to the LAMV prototype strain, the conserved cysteine residues found in most of the other flaviviruses including six cysteines in the preM, 12 in the E protein and 12 in the NS1 protein (Chambers et al., 1990a) were also found in LAMV_M07 and ILOV. The potential N-glycosylation sites in LAMV_M07 included two sites in preM, one site in E and four sites in NS1 protein that were identical to those of the LAMV prototype strain. In contrast to LAMV and LAMV_M07, which had one potential N-glycosylation site in the E protein, none were detected in the ILOV E protein. The putative fusion loop region of E the protein (residues 98–110) in the LAMV prototype and LAMV_M07 strain were found to be similar to those of other flaviviruses. However, in the case of ILOV, residue 110 was an arginine (R), whereas the corresponding residue in most of the other flaviviruses was a lysine (K).

Similar to LAMV and LAMV_M07, four potential N-glycosylation sites were predicted in the ILOV NS1 protein, three initial ones being at the same positions (LAMV polyprotein N884, N897, N981) and the last one being 4 residues before the location of corresponding LAMV N-glycosylation site (LAMV polyprotein N 1081). No DNA sequences corresponding to the genomic RNA of LAMV prototype virus, LAMV-M07 or ILOV were detected in the infected cells using primers targeted to the NS5 gene.

**Fig. 1:**
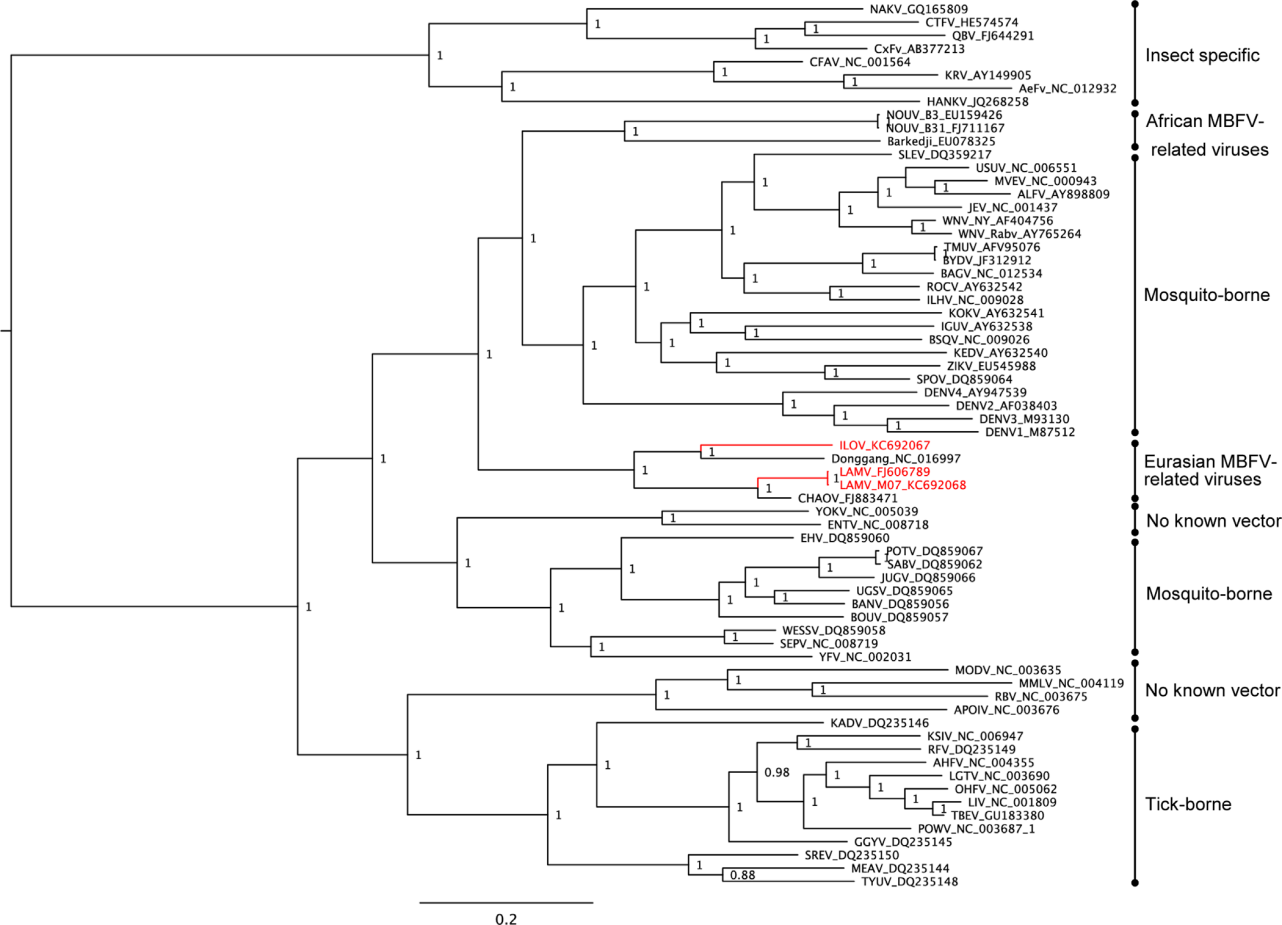
Bayesian phylogenetic tree based on nearly complete polyprotein ORF sequences of flaviviruses (alignment trimmed according to the shortest sequence, Barkedji virus). The novel MBFV-related viruses: Nounane virus (NOUV), Barkedji virus, Ilomants virus (ILOV), Donggang virus, Lammi virus (LAMV) and Chaoyang virus (CHAOV). The African and Eurasian groups are indicated. The Finnish Ilomantsi and Lammi virus strains are indicated in red.

**Fig. 2:**
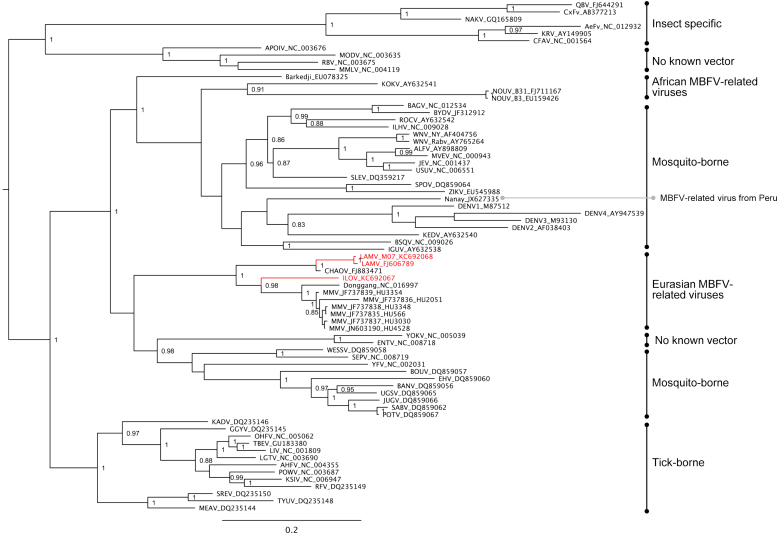
Bayesian phylogenetic tree based on partial NS5 gene sequences available also for Spanish Marisma mosquito virus (MMV) isolates and Nanay virus from Peru.

**Fig. 3:**
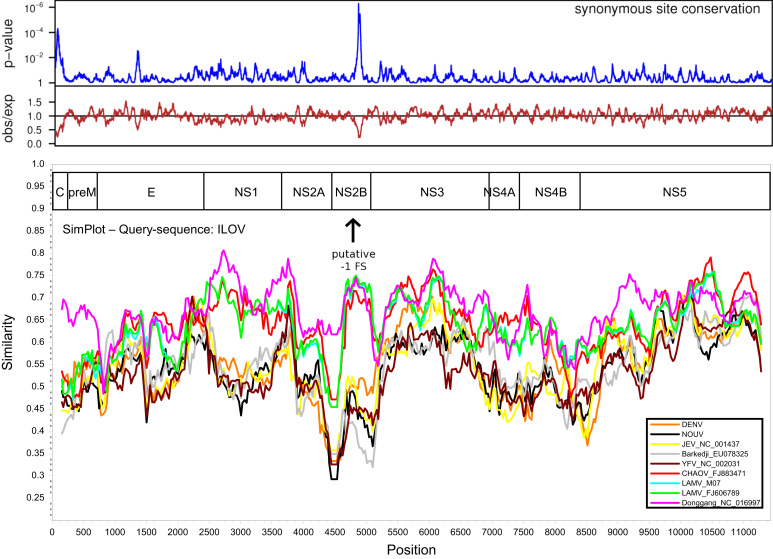
SimPlot analysis and the synonymous site conservation plot of ORF nucleotide sequences. Conservation at synonymous sites in an alignment of ILOV, DONV, LAMV and CHAOV polyprotein coding sequences, using a 15-codon sliding window. The lower panel (brown) shows the ratio of the observed number of substitutions to the number expected under a null model of neutral evolution at synonymous sites, whereas the upper panel (blue) shows the corresponding *p*-value.

**Fig. 4:**
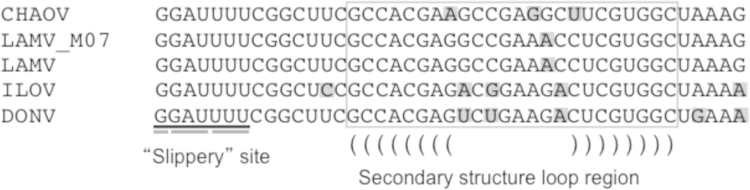
Sequence alignment of the NS2AB region associated with frameshift potential. The “slippery” site is underlined and the predicted 3′ stem-loop structure is boxed. Predicted base-pairings are indicated with parenthesis. Nucleotide variations are indicated with shading.

Novel flavivirus sequences that are related to LAMV and ILOV originating from different geographical locations are documented in public databases (Table 1), including Marisma mosquito virus (MMV) from Spain (Vazquez et al., 2012), Barkedji virus (BARKV) from Senegal (unpublished, GenBank accession EU078325.1) and Israel (Kolodziejek et al., 2013), Nounane virus from Cote d׳Ivoire (Junglen et al., 2009), Donggang virus (DONV) (unpublished, GenBank accession NC_016997), Chaoyang viruses (CHAOV) from China (GenBank accession FJ883471) and South Korea (Lee et al., 2013, Wang et al., 2009) and Nanay virus from Peru (Evangelista et al., 2013). The results of the phylogenetic analysis of the available complete coding sequences in public databases suggest that the MBFV-related viruses form two distinct lineages, which are phylogenetically positioned among the mosquito-borne flaviviruses. In the ORF analysis both of these lineages were positioned between the large cluster of YFV-related viruses (Edge Hill, yellow fever and the Entebbe bat virus group) and the rest of the MBFVs including dengue virus and the JEV complex. NOUV and BARKV were grouped forming one lineage whereas LAMV and ILOV were separated from these viruses and grouped with Chinese viruses CHAOV and DONV (Fig. 1). As the ORF sequences were not available for all the related MBFV-associated viruses, a separate analysis was based on partial NS5 gene sequences (Fig. 2). This additional analysis supported the close association of LAMV and ILOV to DONV and CHAOV, and furthermore showed that the Spanish MMV isolates also belonged to this group of Eurasian viruses. However, in this tree BARKV was separated from NOUV, which was closely associated with KOKV. Additionally NANV, which was previously shown to group with NOUV (Evangelista et al., 2013), was instead associated with the DENV group. The divergence of the viruses of Eurasian origin from those originating in Africa was also apparent in the ORF SimPlot analyses (Fig. 3). The Eurasian viruses (LAMV, LAMV-M07, CHAOV, DONV) showed greatest similarities to ILOV, particularly in the NS1, NS2B, NS3 and NS5 genes. The greatest level of similarity was between ILOV and DONV in the NS1 gene, approximately 80%. The sequence elements associated with ribosomal frameshifting, the G_GAU_UUU “slippery” site (underscores separate polyprotein-frame codons) and the presence of a 3′ adjacent predicted RNA stem-loop structure as previously identified in the NS2A/NS2B coding sequence of CHAOV and LAMV (Firth et al., 2010), were found to be strictly conserved in the Eurasian virus group (Fig. 4). In a comparison of the ILOV, DONV, LAMV and CHAOV polyprotein coding sequences, the sequence regions including the frameshifting associated elements were found to coincide with the region of highest conservation at synonymous sites (Fig. 3), thus indicating that purifying selection operates to preserve more than just the NS2B amino acid sequence in this region.

**Table 1:** Mosquito-borne flavivirus-related viruses.

**Virus**	**Strain or isolate designation**	**GenBank accession**	**Isolation source**	**Country of origin**	**Year**	**Available sequence data**	**Culture experiments**		**Refs.**
							**In vitro culture in vertebrate cells**	**In vivo infection experiments**	
Nounane virus (NOUV)	B31	FJ711167	*Uranotaenia mashonaensis*	Cote d’Ivoire	2004	ORF	Not successful (Vero, BHK, 293, A549, Hep2, PSEK, chicken embryo fibroblast)	No information	Junglen et al. (2009)
	Nounane_B3	EU159426							
Barkedji virus (BARKV)	ArD86177	EU078325	No information	Senegal	–	Nearly complete ORF	No information	No information	Unpublished
	363/11	KC496020	*Culex perexiguus*	Israel	2011	Nearly complete ORF	Not successful (Chicken embryo fibroblast DF-1, Vero)	No information	Kolodziejek et al. (2013)
Chaoyang virus (CHAOV)	Deming	FJ883471	*Aedes vexans*	China	2008	ORF	No information	No information	Wang et al. (2009) (In Chinese)
	HLD115	NC_017086	Mosquito		2010	ORF	No information	No information	Unpublished
	BeiBei	FJ812035	Mosquito		2008	E			
	ROK144	JQ068102	*Aedes vexans nipponii*	Republic of Korea	2003	ORF	Not successful (BHK, primary duck, primary chicken, Vero)	No information	Lee et al. (2013)
Donggang virus (DONV)	DG0909	NC_016997	*Aedes* spp.	China	2009	ORF	No information	No information	Unpublished
Marisma mosquito virus (MMV)	HU4528/07	JN603190	*Ochlerotatus caspius*	Spain	2007	NS5, partial	Yes, viral RNA and CPE on Vero and BHK-21, but one passage only	No information	Vazquez et al. (2012)
	HU3348/06	JF737838			2006			No information	Vazquez et al. (2012)
	HU2051/05	JF737836			2005			No information	Vazquez et al. (2012)
	HU3354/06	JF737839			2006			No information	Vazquez et al. (2012)
	HU3030/06	JF737837			2006			No information	Vazquez et al. (2012)
	HU566/03	JF737835			2003			No information	Vazquez et al. (2012)
Nanay virus (NANV)	PRD316/PER/09	JX627334 (E)	*Culex melanoconion*	Peru (Iquitos)	2009	E, NS5 (partial)	Not successful (Vero-76, Vero-E6, BHK, LLCMK, MDCK, A549, RD)	Not successful (Suckling mice)	Evangelista et al. (2013)
		JX627335 (NS5)							
Lammi virus (LAMV)	LAMV	FJ606789	*Aedes cinereus* (COI based identification)	Finland 2004		ORF	Not successful (primary chicken, SH-SY5Y, HEK293, HeLa, Neuro2A, BHK-21, PK-15, Vero, VeroE6, XTC, BGM, Hep, MA104, L929, MRC-5, SW13, MCDK)	Not successful (Suckling mice)	Huhtamo et al. (2009), This article
	LAMV-M07	KC692068	Mosquito pool, likely *Aedes and/or Ochlerotatus* spp. (COI based identification)	Finland 2007		ORF	Not tested	Not successful (Suckling mice)	This article
Ilomantsi virus (ILOV)		KC692067	Mosquito pool, likely *Ochlerotatus riparius* and/or *Anopheles* spp. (COI based identification)	Finland 2007		ORF	Not successful (Vero, VeroE6, XTC, BGM, Hep, MA104, L929, MRC-5, SW13, HEK293, BHK-21. MCDK)	Not successful (Suckling mice)	This article

The genome termini untranslated sequence (UTR) amplification and sequencing was successful only for the prototype LAMV strain and resulted in 96 bp for the 5′ UTR and 326 bp for the 3′ UTR. The LAMV UTR sequences were aligned and compared to those of CHAOV, DONV, YFV, DENV-1–4, JEV and WNV UTRs (not shown). The LAMV genome ends included the dinucleotides conserved in the entire genus *Flavivirus*, 5′-AG and 3′-CU. The conserved 3′ end dinucleotide CU was also present in CHAOV and DONV sequences. Unlike in LAMV, the DONV and CHAOV 5′ UTRs did not start with 5′-AG, but instead an AG dinucleotide was located as 5th and 6th nucleotide in their sequences. Additionally, sequences resembling those of MBFV conserved 5′ cyclization sequences were identifiable in C gene regions of LAMV, CHAOV and DONV. The 3′ UTR conserved consensus sequences of CS1 and CS2 found in all MBFV, with a few nucleotide differences, were also observed in LAMV, CHAOV and DONV sequences. However, repeated RCS2, CS3, RCS3 sequences typical for JEV and DENV groups, or tandem repeats (additional to CS1 and CS2) characteristic for the YFV group were not identified in these three viruses. No poly A tracts were found in the 3′ UTRs of LAMV, CHAOV and DONV whereas all three had the mosquito and tick-borne flavivirus conserved pentanucleotide sequence (CACAG) (Wengler and Castle, 1986) located near the end of the 3′ UTR.

### *In vitro* and *in vivo* culture experiments

In common with the previous experiments on LAMV, the new isolates, LAMV-M07 and ILOV, did not cause disease in mice, and no evidence for viral replication was found by RT-PCR analysis of RNA extracted from mouse brains. Only mosquito cells (C6/36, AE, AA23, A20) showed CPE, viral RNA and antigen production after infection with LAMV or ILOV. None of the infected cell lines of various vertebrate cell lines at different culture temperatures developed visible CPE. Furthermore no viral antigens were detected in IFA and no increase of viral RNA was seen in the culture supernatant after incubation. As the culture conditions may affect *in vitro* host range as documented for the Rabensburg strain (RABV) of West Nile virus which requires a lowered culture temperature for vertebrate cell infectivity (Aliota and Kramer, 2012) different culture temperatures were tested for LAMV and ILOV (37 °C, 33 °C, 27 °C). However LAMV and ILOV did not replicate in vertebrate cells regardless of the temperature used. Temperatures that resulted in replication of LAMV and ILOV in C6/36 cells included room temperature (approx. +22 °C), 27 °C and 30 °C. No replication of LAMV and ILOV was observed in infected C6/36 cells at 33 °C or 37 °C.

### Immunofluorescence assay

The LAMV and ILOV antigens were identified in the infected C6/36 cells using monoclonal antibodies known to be flavivirus-group reactive (*n*=2) (Supplementary Fig. 1). Additional tests revealed that flavivirus antibody-positive human sera were reactive with LAMV and ILOV antigens, including pooled WNV-positive human sera (*n*=4) and human sera from patients known to have been infected with any one of the four DENV serotypes (*n*=9) (Huhtamo et al., 2010). The antibody tests suggested that ILOV was more antigenically reactive with the flavivirus-antibody-positive human sera than LAMV as determined by serial serum titration comparisons (Supplementary table). None of the tested antibodies were reactive with the insect-specific HANKV-infected C6/36 cells or non-infected C6/36 cells.

### Mosquito vector identification

Sequences obtained from the cloned mosquito pool PCR products showed that the virus-positive pools contained several mosquito species. The clones sequenced from the two LAMV-M07 positive pools formed nine individual consensus sequence (GenBank accession numbers KC778408, KC778410, KC778411, KC778412). In the phylogenetic analysis, these sequences clustered together with sequences of *Ochlerotatus riparius*, *Oc. punctor*, *Oc. annulipes* and *Oc. cantans* (Supplementary Fig. 2A and B). The sequences of clones from the two mosquito pools positive for ILOV formed four consensus sequences (GenBank accession numbers KC778408, KC778410, KC778411, KC778412) that grouped with *Oc. riparius* (Supplementary Fig. 2A) and three sequences that grouped with *Anopheles* sequences that were used as an outgroup for the analysis (data not shown).

## Discussion

During recent years cell culture virus isolation and molecular screening of mosquitoes for flaviviral pathogens has resulted in the isolation and detection of numerous novel flaviviruses from various locations that seem to be specific for mosquito hosts. These include two distinct genetic groups: the CFAV-related viruses designated as insect-specific flaviviruses (ISFs) that appear to be ubiquitous in mosquitoes (Cook et al., 2012) and have also been detected in sandflies (Moureau et al., 2010). These viruses have been detected in most regions of the world, including Finland (Cook et al., 2012, Huhtamo et al., 2012). The second group includes mosquito-borne flavivirus-related viruses that have been detected in Europe (Huhtamo et al., 2009, Vazquez et al., 2012), Asia (Lee et al., 2013, Wang et al., 2009), Africa (Junglen et al., 2009), the Middle East (Kolodziejek et al., 2013) and South America (Evangelista et al., 2013).

In this study, strains of MBFV-related viruses were isolated from mosquitoes collected in 2007 in eastern Finland that represent strains of previously isolated LAMV and a potentially novel flavivirus, tentatively designated Ilomantsi virus (ILOV). The isolation of several strains of LAMV and ILOV from a relatively small number of mosquitoes (*n*≈1400), and some strains from the same locations, suggest co-circulation of the two viruses and presumably indicates their high prevalence in local mosquitoes. As the mosquito collection locations in eastern Finland were approximately 400–500 km distant from the site where the prototype LAMV was originally isolated in southern Finland, it can reasonably be assumed that LAMV is widespread in Finland and possibly also elsewhere in northern Europe.

Based on sequence comparisons, we were able to identify a total of eight distinct viruses as MBFV-related viruses including the tentative novel species described here, ILOV, LAMV, NOUV, BARKV, CHAOV, DONV, NANV and MMV. From the representative viruses, for which the complete genome sequences, including UTRs are currently available (LAMV, CHAOV, DONV) it can be concluded that the genome organization and conserved sequence elements in the UTRs of these viruses resemble those of mosquito-borne flaviviruses. The available sequence data suggest that the individual MBFV-related viruses originating from different geographical locations are divergent and thus may have different biological properties. The sequence analysis presented here included all MBFV-related viruses described to date. Separate analyses were performed for the available ORF data and partial NS5 data available for all MBFV-related viruses. The results demonstrated that the phylogenetic associations of the individual viruses could change when more sequence data were included in the analysis. Previously LAMV was associated with African NOUV, in contrast to the current analysis where a group of Eurasian viruses was identified including LAMV, CHAOV, ILOV, DONV and MMV. The Eurasian group was clearly separated from viruses originating from Africa, the Middle-East or South America (NOUV, BARKV, NANV) in both ORF and NS5 gene analyses. Previously NOUV and NANV (Evangelista et al., 2013) and NOUV and BARKV (Kolodziejek et al., 2013) were phylogenetically associated. In the current analysis of ORF sequences available for NOUV and BARKV, these were grouped together. However, in the NS5 tree these viruses were positioned differently, NOUV associated more closely to KOKV whereas NANV was closer to the DENV group. Clearly, complete genome sequence data of all these viruses will be necessary before their phylogenetic relationships and taxonomic status can be reliably determined.

The recently identified frameshifts of some mosquito-borne and other mosquito-hosted flaviviruses, and the resulting protein products (Blitvich et al., 1999, Firth and Atkins, 2009, Firth et al., 2010, Melian et al., 2010) may play a significant role in modulating the biological properties of these viruses. The absolute conservation of the “slippery” heptanucleotide G_GAU_UUU in the NS2B sequences of the European and Asian MBFV-related viruses, including CHAOV, LAMV, ILOV and DONV, suggests that it may have a biological function. Identical shifts sites (i.e. G_GAU_UUU) have been identified also in the newly described insect-specific *Mesoniviridae* (Lauber et al., 2012; Zirkel et al., 2013) and the insect-specific flaviviruses KRV and AEFV, besides some species of *Umbravirus*, *Dianthovirus* and *Totivirus*. Unfortunately, the complete genomic sequences of MMV and NANV are not currently available for comparison in this context. Nevertheless, based on our knowledge of the taxonomically recognized arthropod-borne flaviviruses it is known that the NS2B protein is a membrane-anchoring subunit of the flaviviral NS2B–NS3 protease complex that is required for catalytic activity (Falgout et al., 1991) in proteolytic processing of the polyprotein precursor (Cahour et al., 1992, Chambers et al., 1990b). Two consecutive regions of NS2B have been shown to be crucial for these processes (Chappell et al., 2008) and the “slippery” site slightly overlaps the end of the latter of these NS2B regions. Thus, the potential frameshift would likely change only a few amino acids of the cofactor domain, but would replace the C-terminal membrane domain (Brinkworth et al., 1999, Droll et al., 2000). Experimental evidence using virus-infected cells will be necessary to demonstrate whether or not the frameshift occurs, and if an elongated form of NS2B protein is formed in these viruses, as previously predicted for LAMV and CHAOV (Firth et al., 2010).

The MBFV-related viruses have been isolated from various mosquito species, but whether these are the only hosts of these viruses is currently unknown. Two phylogenetically associated viruses, African NOUV and Peruvian NANV were isolated from different mosquito species: NOUV from *Uranotaenia* (*Pseudoficalbia*) *mashonaensis* (Junglen et al., 2009) and NANV from *Culex* (*Melanoconion*) *ocossa* (Evangelista et al., 2013). Although the vector for BARKV in Senegal is unknown, it was hosted by *Culex* (*Culex*) *perexiguus* in Israel (Kolodziejek et al., 2013). The available information for the Eurasian viruses indicates involvement of various *Ochlerotatus* species (Huhtamo et al., 2009, Lee et al., 2013, Vazquez et al., 2012, Wang et al., 2009) although the CHAOV isolate ROK144 RNA was also detected in *Culex* (*Culex*) *pipiens* and *Armigeres* (*Armigeres*) *subalbatus* (Lee et al., 2013). *Ochlerotatus* mosquitoes were likely to be the hosts for the LAMV-M07 strain and ILOV, although further studies are needed for reliable identification of the host or vector species of ILOV and LAMV as the current host species identification may be biased due to mosquito pooling and retrospective COI-based identification.

Unlike the MBFVs that generally infect various mosquito and vertebrate cell lines, in addition to newborn mice (Karabatsos, 1985, Kuno, 2007), none of the MBFV-related viruses have a known vertebrate host and the experimental *in vivo* infections (LAMV, LAMV-M07, ILOV, NANV) did not result in visible disease in newborn mice. Only MMV has been reported to infect transiently Vero and BHK cells (Vazquez et al., 2012) but further experiments are needed to show whether or not MMV and other MBFV-related viruses could adapt to vertebrate cells. Although transient *in vitro* replication of vector-borne flaviviruses in anomalous vectors has been considered as evidence of past host association and possible vector-group shift in the evolution of these viruses (Kuno et al., 1998, Kuno, 2007) in the absence of further data, the possible implications of transient *in vitro* replication in potential vertebrate hosts is not clear. All the previously characterized flaviviruses phylogenetically associated with the cluster of mosquito-borne flaviviruses, except for the MBFV-related viruses discussed here, do replicate in vertebrate cells (Kuno, 2007). In light of this, the apparent inability of LAMV and related viruses to infect vertebrate cells is a unique property. The other exceptional viruses in the mosquito-borne flavivirus group are the viruses with no mosquito vector associations, Cacipacore, Entebbe bat, Sokuluk and Yokose viruses which however can be *in vitro* cultured in mosquito cells (Kuno, 2007).

Further characterization is required before the diverse group of MBFV-related viruses can be absolutely determined to be mosquito-specific and nonpathogenic for humans and other vertebrates. As there does appear to be a likelihood of superinfection of mosquito hosts in areas of co-circulation of MBFV and MBFV-related viruses, it will therefore be important to establish whether or not co-infection between MBFV and MBFV-related viruses could affect the replication and epidemiology of the pathogenic flaviviruses. We conclude that although the novel MBFV-related viruses are likely to be mosquito-specific, their genetic and antigenic associations with the MBFVs clearly differentiate them from the ISFs, and the two clades are clearly genetically separate groups of flaviviruses. Our current understanding of the variety of different flaviviruses and their attributes, including the evolutionary origins of flaviviruses, is potentially biased by the small number of representatives of different lineages that to date mainly comprise viruses associated with human disease and those easily cultured in vertebrate cells or in suckling mouse brains. The characterization of novel flaviviruses and sequencing of their complete genomes will enable a more robust analysis of the evolutionary history of flaviviruses, including correlation of genetic lineages with biological properties. Importantly, since serological cross-reactions have been detected, specific methodologies will need to be developed for the distinction of these MBFV-related viruses from the other currently recognized flaviviruses.

## Materials and methods

### Mosquito collection and virus isolation

Mosquitoes were collected near the end of the mosquito season in August 2007 using hand nets in two areas approximately 200 km apart in eastern Finland, around Sotkamo (N64°08′, E28°23′) and Ilomantsi (N62°46′, E30°59′) located near the Russian border. The mosquitoes (*n*≈1400) were placed in Eppendorf tubes, 10 mosquitoes/pool and homogenized using sterile sand in Dulbecco׳s PBS supplemented with 0.2% BSA and antibiotics. Two pools were combined for each virus isolation attempt. In total, 68 isolation attempts were performed in both C6/36 (ATCC CRL-1660) and Vero cells (ATCC CL-81) grown in 25-cm^2^ culture flasks. The mosquito homogenates were inoculated unfiltered onto Vero cells and filtered through 0.45 nm filters prior to inoculation onto C6/36 cells. Cells were observed for cytopathic effects (CPE), and cells and supernatant media were harvested when approximately 50% of the monolayer showed CPE. When no CPE was observed, the cells and supernatant media were harvested on day 14 post-inoculation. All infected cells were studied via indirect immunofluorescence assay (IFA) as previously described (Huhtamo et al., 2009).

### Immunofluorescence assay

C6/36 cells infected with LAMV, ILOV and HANKV were washed 3 times in phosphate buffered saline (PBS), air-dried on microscope slides and fixed in cold acetone for 7 min. Primary antibodies were diluted in PBS and incubated on slides for 1 h at 37 °C and followed by washing 3 times in PBS and once in distilled water. Appropriate FITC-conjugated secondary antibody, either anti-human-IgG (Jackson Immunoresearch) or anti-mouse-immunoglobulins (DAKO) was diluted in PBS according to manufacturer׳s instructions and incubated on air-dried slides for 30 min and washed as previously. The cells were examined for the presence of viral antigens using a fluorescence microscope. The primary antibodies included flavivirus-specific monoclonal antibodies HB-112 (Henchal et al., 1982), 813 (Gould et al., 1985), pools of WNV-positive human sera from the European Network for Diagnostics of Imported Viral Diseases (ENIVD) (*n*=4) and a set of 9 human serum samples from characterized dengue patients infected with DENV-1–4 (DENV-1: *n*=2; DENV-2: *n*=1, DENV-3: *n*=5; DENV-4: *n*=1) (Huhtamo et al., 2010). The tested human serum samples were titrated on virus antigen slides for comparing the reactivity with the different flaviviral antigens. The HANKV ISF (Huhtamo et al., 2012) and negative C6/36 cells were included as controls in all antibody tests.

### Electron microscopy

Virus samples for electron microscopy were concentrated from the supernatant medium of C6/36 inoculated cells using Amicon 100 kDa concentrator columns and placed on copper grids prior to negative staining.

### Genome amplification and sequencing

Viral RNA was extracted from the supernatant medium of IFA-positive virus isolation cell cultures using the QiaAmp viral RNA mini kit (Qiagen) according to the manufacturer׳s instructions and used as a template in RT-PCR tests. Initially, partial NS3 genes were amplified and sequenced from all isolates using previously published pan-flavivirus primers (Grard et al., 2007). Additionally, for two isolates (M0719, M0724), partial NS5 (Scaramozzino et al., 2001) and E genes (Gaunt and Gould, 2005) were also amplified and sequenced. The derived sequences were used for designing the primers for long PCR (primer sequences available from the authors upon request) producing overlapping amplicons to cover the remaining fragments of the genomes. These were amplified using the cMaster RT plus PCR System kit (Eppendorf) and the complete open-reading frame sequences were determined using the LoPPS method (Emonet et al., 2007). The UTR sequence determination was undertaken using the 5′/3′ RACE kit (Roche) and the products were T-A cloned to pGEM-T vector (Promega) and sequenced. Data were combined to derive consensus sequences using Sequencher v.4.8 (Gene Codes).

To test whether the LAMV prototype strain and the isolates M0719 and M0724 produced DNA forms of the genomic RNA, DNA was extracted from infected C6/36 cells using TriPure Isolation reagent (Roche) and treated with RNAse A (Ambion) prior to testing in direct PCR using pan-flavivirus primers (Scaramozzino et al., 2001).

### Sequence analysis

The potential N-glycosylation sites were predicted for LAMV_M07 and ILOV proteins using the NetNGlyc 1.0 server (available at http://www.cbs.dtu.dk/services/NetNGlyc/). Protein sequence alignments were prepared using MUSCLE (Edgar, 2004). Nucleotide sequences were then aligned based on the coding regions of the aligned polyprotein sequences using the program EMBOSS tranalign (available at http://emboss.bioinformatics.nl/cgi-bin/emboss/tranalign). Alignments were submitted to the GBlocks program, which objectively eliminates poorly aligned positions and divergent regions (Castresana, 2000). As the Barkedji virus (BARKV) sequence (EU078325.1) was the only incomplete sequence used in the open reading frame (ORF) alignments, these were trimmed to match this sequence, lacking 19 amino acids from the beginning of the C gene. Additional analysis was performed for partial (935 bp) NS5 gene sequences. The NS5 alignment was trimmed according to the shortest Spanish MMV isolate sequences. The ORF nucleotide sequence similarity plots were generated via the SimPlot program version 3.5.1 (Lole et al., 1999) using a sliding window of 300 bp. Synonymous site conservation was calculated as described previously (Firth et al., 2011).

A Bayesian estimation of the phylogeny was performed using MrBayes v 3.1.2 (Huelsenbeck and Ronquist, 2001, Ronquist and Huelsenbeck, 2003). For all analyses, the Bayesian Markov chain Monte Carlo (MCMC) method was implemented (Huelsenbeck and Ronquist, 2001). All parameters were estimated from the data under default priors and Markov chains were run for a minimum of 20 million generations, with the first 10% of samples discarded as burnin. Support for nodes was assessed using posterior probability values calculated in MrBayes. All phylogenetic analyses were carried out on the freely available Bioportal server (www.bioportal.uio.no). Stationarity was assessed at effective sample sizes (ESS)>400 using Tracer v1.4.1 (Drummond and Rambaut, 2007).

### *In vitro* and *in vivo* culture experiments

Cell lines from different vertebrate species including monkey (Vero, Vero E6, BGM, MA104), human (Hep, MRC-5, SW13, HEK293), mouse (L929), hamster (BHK-21) canine (MCDK), toad (*Xenopus laevis*, XTC) and snake cells (*Boa constrictor*, Hetzel et al., 2013) and mosquito cell lines (C6/36, AE, AA23, A20) were experimentally infected using the supernatant media from LAMV prototype strain and ILOV infected C6/36 cells and observed for cytopathic effects daily. Mosquito cells and *Xenopus* (toad cells -XTC) were grown in L-15 medium. SW13 cells and HEK293 were grown in RPMI media, MRC-5 in BME and the other vertebrate cell lines in MEM. All mammalian cells were incubated at 37 °C for 7 days. After incubation with virus inoculum overnight, the cells were thoroughly rinsed three times with PBS before adding new media. Immediately after adding the fresh media, a 0-sample was taken from the supernatant, and after 7 days of incubation when also the infected cells were harvested and studied in IFA for viral antigens. Additionally HEK293, BGM, Vero, XTC, snake cells and C6/36 cells were tested at 37 °C, 33 °C and 27 °C, and XTC, snake and C6/36 at 30 °C by infecting them for 1 h with virus inoculum following rinsing and adding new media as described above. Supernatant samples were collected immediately after adding of new media and after 3, 7 and 10 days of incubation. Total RNA was extracted from the supernatant medium samples using the EZ1 Biorobot and the Qiagen virus mini kit or using the Qiagen QIAamp viral RNA Mini Kit and examined for flaviviral RNA using real-time RT-PCR (Moureau et al., 2007). The infected cells were further studied for flaviviral antigens in IFA using flavivirus-specific antibodies (Henchal et al., 1982) as described previously (Huhtamo et al., 2009).

The original supernatant media obtained from LAMV prototype virus, LAMV-M07 strain and ILOV isolate from infected C6/36 cells were studied for their ability to infect 0–2 days old NMRI mice (Animal experiment license ESLH-2008-06558) following intracerebral inoculation. The mice were observed daily for clinical symptoms and sacrificed on day 14 p.i. Total RNA was extracted from the brain tissues using TriPure isolation reagent (Roche) and tested for the presence of flaviviral RNA by RT-PCR (Scaramozzino et al., 2001).

### Mosquito vector identification

Retrospective identification of the mosquito species present in virus-positive pools was conducted as per Huhtamo et al. (2012). Briefly, DNA present in the mosquito homogenate pools (*n*=4) was extracted using DNeasy Blood and tissue kit (Qiagen) and used as a template for PCR using primers targeted to the cytochrome oxidase 1 (COI) gene region commonly referred to as the “barcode” region (Folmer et al., 1994). The PCR products were cloned into pGEM-T vector (Promega) and a total of five individual clones were sequenced from each of the four PCR products representing the virus-positive mosquito pools.

A total of 20 individual clones was sequenced and found to represent nine distinct sequences for mosquitoes contributing to the LAMV-M07-positive pools, namely LAMV_M07_Contig 1 to Contig 9 inclusive, and seven distinct sequences for mosquitoes contributing to ILOV-positive pools, namely ILOV_Contig 1 to Contig 7 inclusive. These COI sequences, originating from mosquitoes from viral-positive pools, were inserted into a bespoke reference backbone data set of nucleotide sequences that included 530 sequences (636 bp) from across the Culicidae in which each species was represented, in general, by multiple individuals. Sequences were aligned using MUSCLE (Edgar, 2004) and a neighbor-joining tree was generated in PAUP⁎ (Swofford, 2000) (data not shown). A smaller “Aedini-subset” alignment was also prepared. Included were (i) all sequences available for those aedine species recorded from Finland (Utrio, 1979), (ii) all other publicly available aedine species with COI sequences that were relevant as indicated by default searches conducted in BLAST and BOLD resources and (iii) sequences from a number of aedine specimens present in the collections at the Natural History Museum, London (data not shown). Finally, two subset alignments were prepared for clarity. For all subset analyses, MODELTEST (Posada and Crandall, 1998) was used to select the best-fit model of nucleotide substitution (the GTR+Γ_4_+I model) and the Bayesian Markov chain Monte Carlo (MCMC) method was implemented in MrBayes v3.1.2. with methodology equivalent to that conducted for viral analyses (Huelsenbeck and Ronquist, 2001).

## Appendix A Supplementary materials

**Figure 1.** Immunofluorescence images of A) ILOV infected C6/36 cells; B) LAMV infected C6/36 cells; C), HANKV infected C6/36 cells; D), uninfected C6/36 cells; E) positive controls: JEV infected Vero E6; F) DENV-3 infected Vero E6 cells. Staining using flavivirus group specific monoclonal antibody HB-112 (Henchal et al., 1982) and anti-mouse FITC-conjugate.”

**Figure 2. A)** Subset of mosquito phylogeny including four cloned sequences from LAMV_M07 mosquito pools (Contigs 6-9) and four cloned sequences from ILOV mosquito pools (Contigs 1-4).

**Figure 2. B)** Subset of mosquito phylogeny including five cloned sequences from LAMV_M07 mosquito pools.

**Supplementary Table 1.** Immunofluorescence assay titration of flavivirus antibody-positive human sera. The IFA titers are given as reciprocal to the last positive dilution. ND-not done.

## Footnotes

Appendix A Supplementary data associated with this article can be found in the online version at doi:10.1016/j.virol.2014.07.015.
