# Supplementary figures and images for: Novel flaviviruses from mosquitoes: Mosquito-specific evolutionary lineages within the phylogenetic group of mosquito-borne flaviviruses
Source: Virology. 2014 Sep;464-465:320–9. doi: 10.1016/j.virol.2014.07.015 (PMC4170750; doi:10.1016/j.virol.2014.07.015)

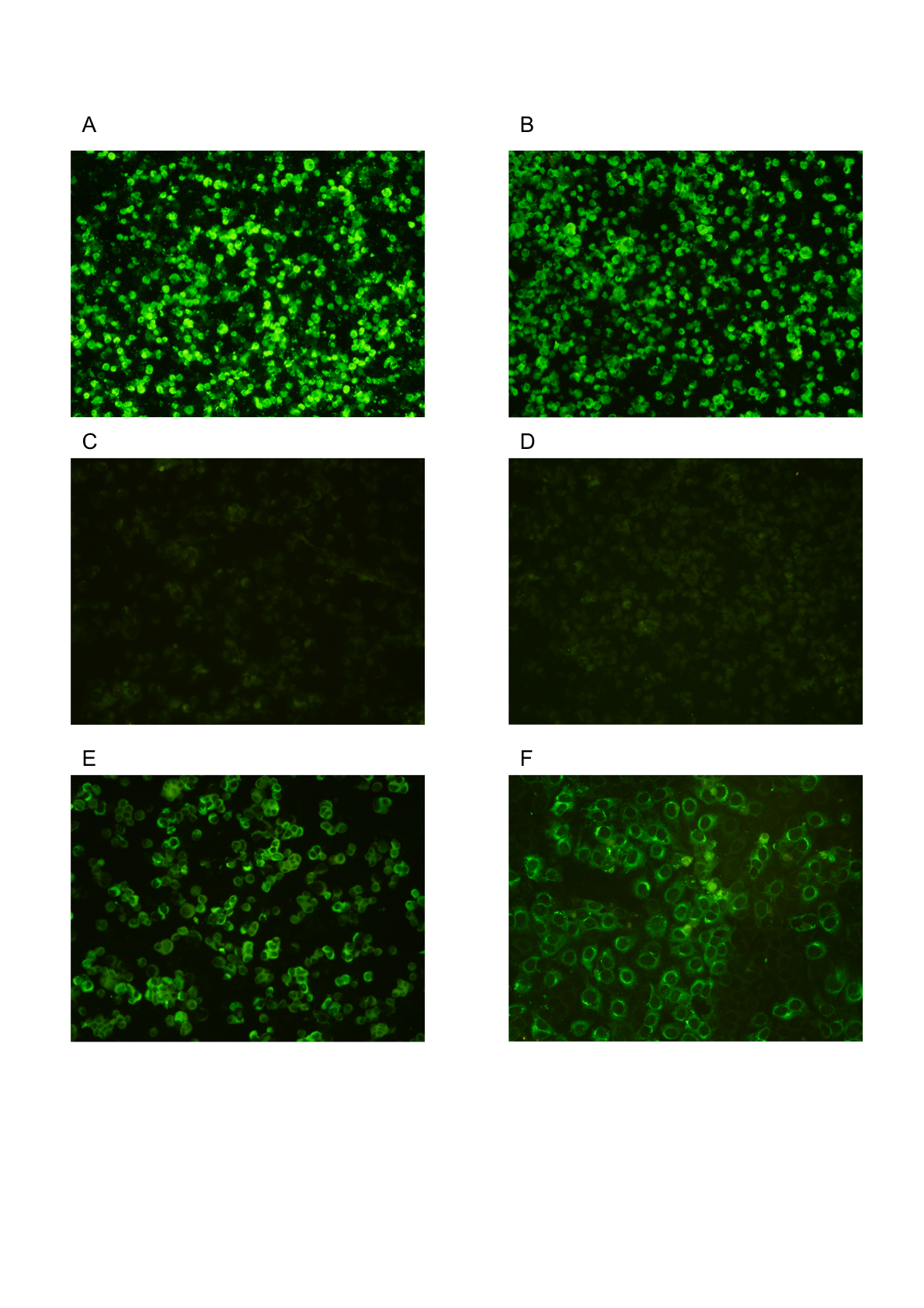

Supplement: Supplementary file 1 — Figure 1. Immunofluorescence images of A) ILOV infected C6/36 cells; B) LAMV infected C6/36 cells; C), HANKV infected C6/36 cells; D), uninfected C6/36 cells; E) positive controls: JEV infected Vero E6; F) DENV-3 infected Vero E6 cells. Staining using flavivirus group specific monoclonal antibody HB-112 (Henchal et al., 1982) and anti-mouse FITC-conjugate.” [file mmc1.zip › Suppl_Figure1.tiff]

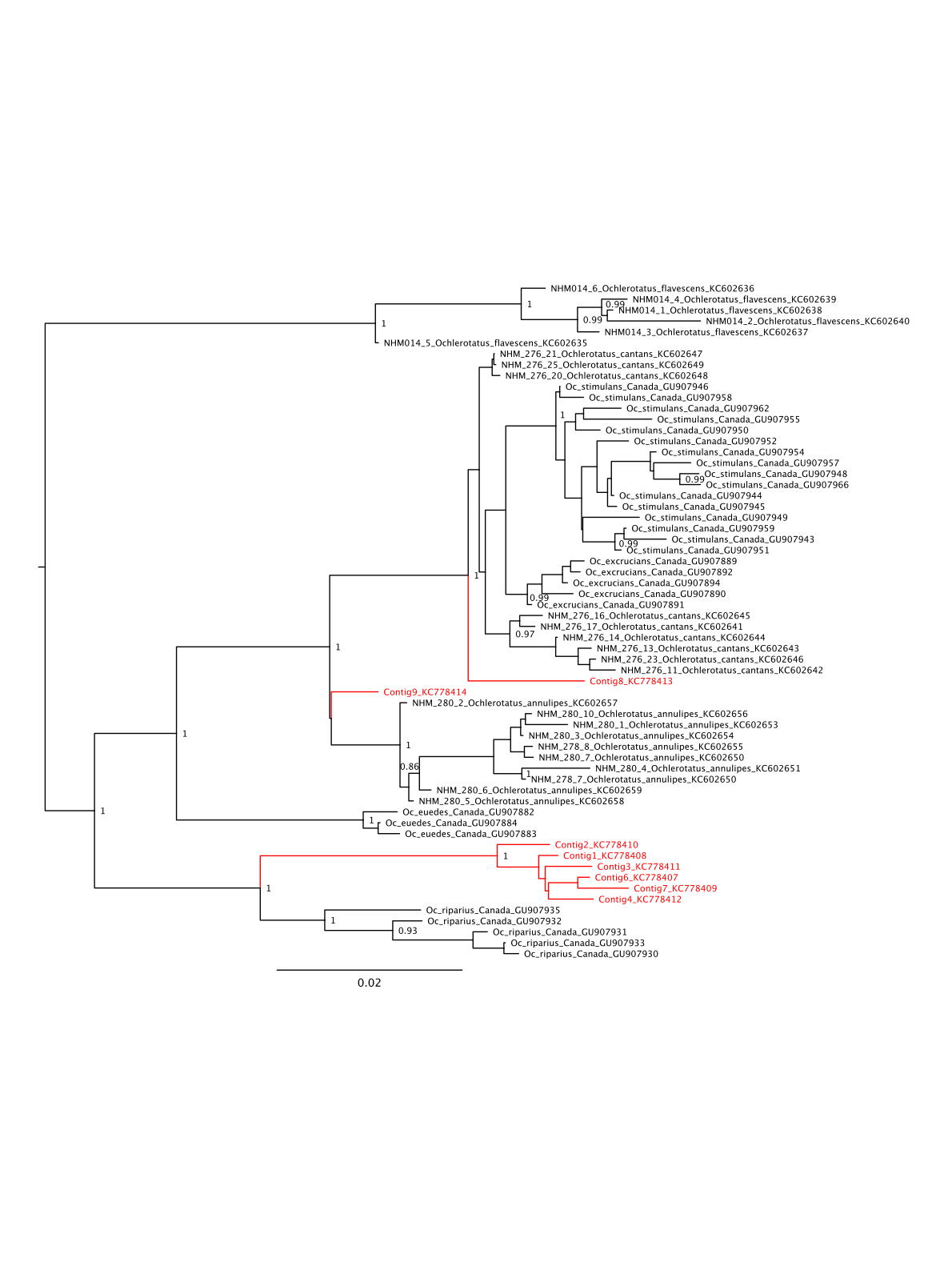

Supplement: Supplementary file 2 — Figure 2. A) Subset of mosquito phylogeny including four cloned sequences from LAMV_M07 mosquito pools (Contigs 6-9) and four cloned sequences from ILOV mosquito pools (Contigs 1-4). [file mmc2.zip › Suppl_fig2A.tiff]

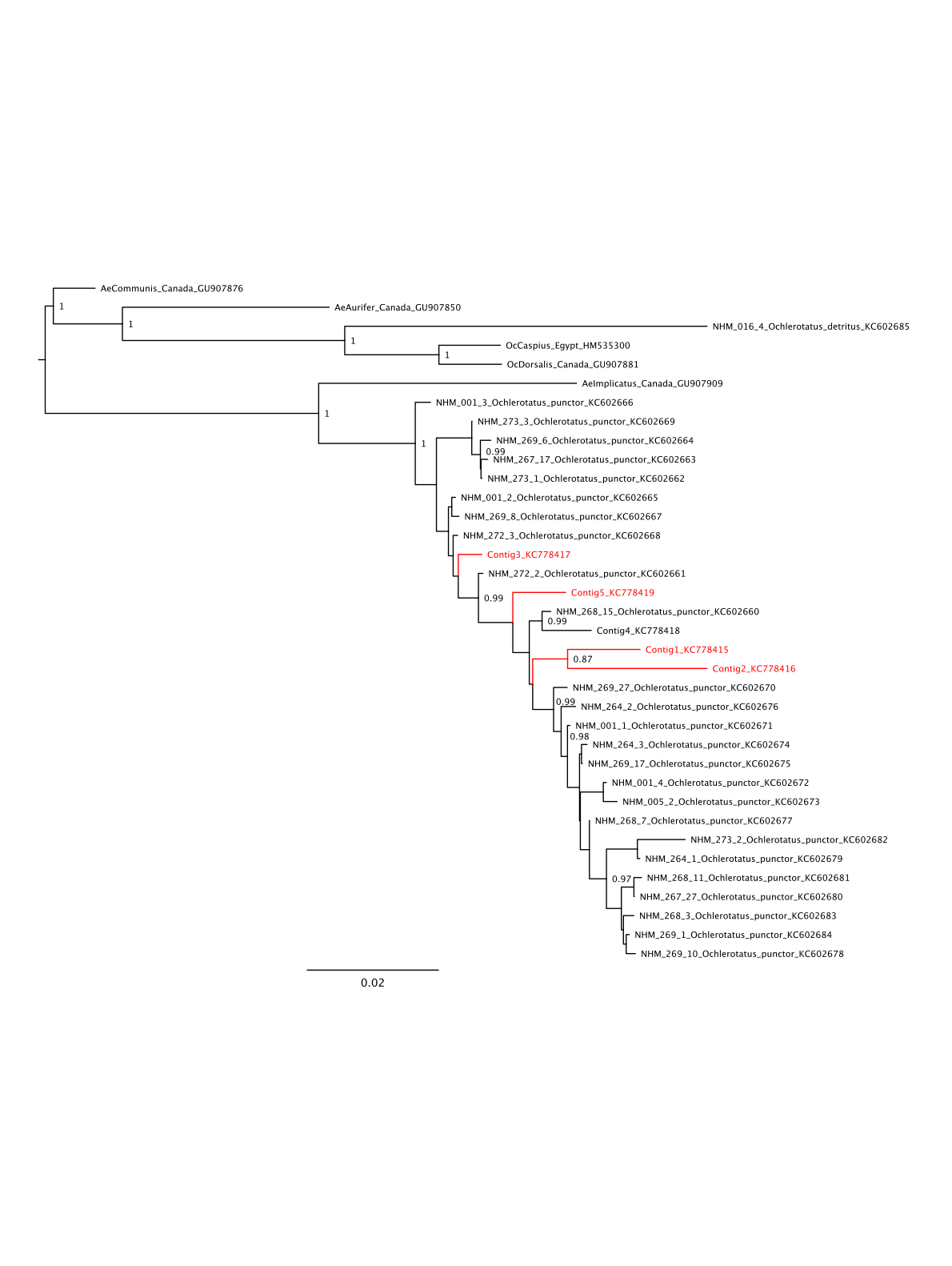

Supplement: Supplementary file 3 — Figure 2. B) Subset of mosquito phylogeny including five cloned sequences from LAMV_M07 mosquito pools. [file mmc3.zip › Suppl_fig2B.tiff]
