# Supplementary material for: Novel flaviviruses from mosquitoes: Mosquito-specific evolutionary lineages within the phylogenetic group of mosquito-borne flaviviruses
Source: Virology. 2014 Sep;464-465:320–9. doi: 10.1016/j.virol.2014.07.015 (PMC4170750; doi:10.1016/j.virol.2014.07.015)
Supplement: Supplementary file 4 — Supplementary Table 1. Immunofluorescence assay titration of flavivirus antibody-positive human sera. The IFA titers are given as reciprocal to the last positive dilution. ND-not done. [file mmc4.doc]

| Virus antigen slide tested | IFA titer | | | | | | | | | | | | |
| --- | --- | --- | --- | --- | --- | --- | --- | --- | --- | --- | --- | --- | --- |
|  | Human serum #1  DENV-4 | Human serum #2  DENV-3 | Human serum #3  DENV-1 | Human serum #4  DENV-3 | Human serum #5  DENV-3 | Human serum #6  DENV-2 | Human serum #7  DENV-1 | Human serum #8  DENV-3 | Human serum #9  DENV-3 | Pooled human sera  BM140375  WNV | Pooled human sera  BM140434  WNV | Pooled human sera  BM141170  WNV | Pooled human sera  BM141410  WNV |
| ILOV C6/36 | 120 | 240 | 20 | 80 | 640 | 640 | 80 | 40 | 80 | 160 | 120 | 320 | 240 |
| LAMV C6/36 | 20 | 60 | >20 | 20 | 80 | 120 | 80 | 20 | 60 | 60 | 20 | 80 | 160 |
| HANKV C6/36 | >20 | >20 | >20 | >20 | >20 | >20 | >20 | >20 | >20 | >20 | >20 | >20 | >20 |
| DENV-3 Vero E6 | 160 | 640 | 160 | 320 | 1280 | 640 | 80 | 80 | 80 | ND | ND | ND | ND |
| JEV Vero E6 | 120 | 240 | 40 | 80 | 1280 | 240 | 80 | 60 | 80 | 240 | 160 | 480 | 480 |
| WNV Vero E6 | ND | ND | ND | ND | ND | ND | ND | ND | ND | <640 | 640 | 480 | 480 |

Supplementary Table 1. Immunofluorescence assay titration testing of flavivirus antibody-positive human sera. The IFA assay titers are given as reciprocal to the last positive dilution. ND-not done.
